# Psychological and Behavioural Within-participant Predictors of Adherence to Oral HIV Pre-Exposure Prophylaxis (PrEP)

**DOI:** 10.1007/s10461-023-04151-8

**Published:** 2023-08-14

**Authors:** Alison Taylor, Rosalie Hayes, Nneka Nwokolo, Gary Whitlock, Olamide Dosekun, Sheena McCormack, Mitzy Gafos, Michael Evangeli

**Affiliations:** 1 Department of Psychology, Royal Holloway University of London, London, UK; 2 Wolfson Institute of Population Health, Queen Mary University of London, London, UK; 3 Chelsea and Westminster Hospital, London, UK; 4 Imperial College Healthcare NHS Trust, London, UK; 5 Medical Research Council Clinical Trials Unit, University College London, London, UK; 6 Department of Global Health and Development, London School of Hygiene and Tropical Medicine, London, UK

**Keywords:** Pre-exposure Prophylaxis (PrEP), adherence, within-participant design, IMB model

## Abstract

Oral PrEP’s effectiveness relies on adequate adherence during periods of substantial HIV risk. Since most PrEP users will miss doses, understanding predictors *within* participants can help to explain adherence. We used a cross-sectional, within-participant design with 67 gay, bisexual, and other men who have sex with men taking PrEP daily. Using a questionnaire, informed by the Information Motivation Behavioral Skills Model, participants were asked about an adherent and a non-adherent episode. PrEP non-adherence was associated with non-normality of the day (p < .001), being out of the home (p < .001), weekend days (p = .01), having company (p = .02), using substances (p = 0.02), not using reminders (p = .03), lower PrEP information (p = .04), lower behavioural skills (p < .001) and less positive affect (p = .002). PrEP adherence assessment could focus on situational variations, supporting the construction of alternative strategies to facilitate adherence in these situations.

**Supplementary Information:**

The online version contains supplementary material available at 10.1007/s10461-023-04151-8.

## Introduction

Gay, bisexual and other men who have sex with men (GBMSM) continue to be disproportionately affected by HIV across the world, and constitute the majority of people diagnosed with HIV each year in many European countries [1]. In the United Kingdom (UK), 36% of new HIV diagnoses were among GBMSM in 2021, predominantly transmitted via condomless anal sex [2]. Since 2015, a year-on-year reduction in HIV diagnoses has been observed among GBMSM in England [2]. This shift has been attributed to combined preventive strategies, including increased testing, earlier initiation of HIV treatment, and the introduction of HIV pre-exposure prophylaxis (PrEP) [3].

PrEP is a relatively novel HIV prevention strategy which refers to the use of antiretroviral therapy (ART) by HIV-negative people to prevent acquisition of HIV. The most commonly used drug combination is oral co-formulated emtricitabine and tenofovir disoproxil fumarate (F/TDF, either as Truvada™ or generic versions) [4, 5]. Until 2020, only oral PrEP was recommended for use by the World Health Organisation (WHO) but more recently the dapivirine vaginal ring and long-acting injectable cabotegravir have been approved [6, 7].

In line with British HIV Association (BHIVA) and British Association for Sexual Health and HIV (BASHH) guidelines [8], GBMSM at elevated risk of acquiring HIV from condomless sex in the UK are offered a choice between two oral PrEP dosing regimens:

1. Daily regimen; one tablet taken every day. Research suggests that for daily oral PrEP to be effective, GBMSM need to take at least four doses a week regardless of sexual activity levels [9].
2. On-demand regimen; two doses of oral PrEP between two and twenty-four hours before sex, a third dose twenty-four hours later and a fourth dose forty-eight hours later [10].

However, the effectiveness of PrEP is compromised by inadequate adherence to either the daily or the on-demand dosing schedule [11–13]. For example, the international iPrEX PrEP randomised control trial observed a 44% reduced risk of HIV acquisition across all participants in the trial arm, yet when analysis compared those with detectable levels of TDF/FTC to those without, this protective effect increased to 92% among those with detectable levels of TDF/FTC [14]. Inadequate adherence may also result in the development of antiretroviral resistance in individuals who unknowingly acquire HIV and continue to take PrEP [12, 15]. Bridging the efficacy-effectiveness gap by understanding and optimising adherence is therefore key to maximising the public health impact of PrEP [16].

Research has highlighted potential *between*-participant predictors of PrEP adherence, including higher levels of HIV risk perception and actual risk behaviour [17–20], less anticipated stigma from disclosing PrEP use [21], behavioural and situational factors (i.e., having an established routine, use of reminders and less travel) [21–24] and increased access to PrEP [25, 26]. However, as with other prophylactic and therapeutic medications [27], there is substantial *within*-participant variation in adherence to PrEP. For example, despite generally high overall adherence to a daily PrEP regimen in the PROUD trial in England, 40% of GBMSM participants did not take their PrEP medication all of the time and 36% intentionally did not adhere for a period [28]. A within-participant approach has been used previously to explore adherence to HIV treatment to identify factors which may vary according to specific contexts, e.g., affect, behaviour and cognition, whilst controlling for static demographic factors [29]. This approach allows more confident causal inferences to be made between potential adherence determinants and medication use, than a between-participants design [30].

The application of a theory to understand PrEP adherence can help identify the active mechanisms underlying this behaviour within specific populations and allow for the development of tailored PrEP adherence interventions [31]. The Information-Motivation-Behavioural Skills (IMB) Model describes behavioural and psychological determinants of HIV-related behaviours [32], and has been specifically adapted to explain ART adherence [33]. The IMB model of adherence behaviour defines ‘information’ as the perceived knowledge about medication use in that situation, e.g., knowing to take medication orally with food. ‘Motivation’ is described as both (i) personal motivation or treatment outcome expectancies and their perceived importance, and (ii) social motivation, or the perception and importance of others’ wishes in relation to adherence. The behavioural skills construct is defined as the objective skills in taking medication as well as perceived self-efficacy in using those skills, e.g., being able to swallow pills and feeling confident in your ability to do so [33]. As it describes motivational and behavioural skills which can change situationally, the IMB model is particularly well-suited to within-participant research. The model also incorporates moderating factors which affect adherence including psychological health, living situation, access to medical care and substance use [34].

Very few studies to date have evaluated the applicability of the IMB model in predicting PrEP adherence between participants. Three studies have found that PrEP adherence is associated with PrEP-related behavioural skills [35–37], with Knox et al. (2022) also finding that PrEP motivation is directly associated with PrEP behavioural skills. Qu et al. (2018) found no association between PrEP adherence and levels of PrEP information, motivation or behavioural skills [38]. Other studies have found empirical support for the IMB model in relation to PrEP uptake, either in terms of willingness to use PrEP [39] or actual PrEP use [40, 41]. However, no study has yet considered the situational psychological and behavioural factors influencing inconsistent adherence *within* individuals. We therefore aimed to investigate within-participant situational differences in adherent and non-adherent episodes in a cross-sectional study, theoretically informed by the IMB model, of adherence episodes. No a priori predictions were made as previous research has not explicitly investigated the within-participant predictors of adherence among PrEP users.

## Methods

### Study Design and Setting

We used a cross-sectional, within-participant study design. Participants were recruited using convenience sampling from two London sexual health clinics who were both participating in the PrEP Impact and DISCOVER trials. The PrEP Impact open-label trial in England aimed to enrol 10,000 participants to address outstanding implementation questions including uptake and adherence to daily and event-based dosing regimens [42]. The Gilead Sciences, Inc DISCOVER multi-national double-blinded randomised control trial aimed to test whether Descovy™ (emtricitabine and tenofovir alafenamide, F/TAF) was as safe and effective as Truvada™ (emtricitabine and tenofovir disproxil fumarate, F/TDF) when used by GBMSM as a pre-exposure prophylaxis within a daily dosing regimen [43].

### Participants

Participants had either enrolled in the PrEP Impact or DISCOVER trials at one of the recruitment sites or attended either site for a general sexual health appointment or monitoring for a private PrEP prescription (either purchased online or in clinic). Eligible participants were HIV-negative GBMSM, at elevated risk of HIV acquisition, over the age of 16, English-speaking, had followed a daily dosing PrEP regimen^1^ for at least three months and had shown inconsistent adherence (i.e., had missed at least one dose) in the previous month.

All recruited participants were approached between September and December 2017, either by their clinician, research nurse, or one of the authors of this study (AT). Once eligibility was determined and consent provided, participants were asked to complete a questionnaire either self-administered online or on paper (with a self-addressed envelope for return) or administered through a Skype or telephone call.

Ethical approval was granted by the London-Surrey Borders NHS Research Ethics Committee and approved by the Health Research Authority in 2017 (REC ref 17/LO/0625; IRAS project ID 224,366).

## Questionnaire Development

Detailed quantitative questionnaire data collection for both a specific adherent and non-adherent episode in the previous month were obtained for each participant. In the absence of a validated within-participant PrEP adherence questionnaire, we adapted the Life Windows Information–Motivation–Behavioral Skills Antiretroviral Therapy Adherence Questionnaire (LW-IMB-AAQ) for PrEP users. This was done through consultation between two of the authors (AT and ME), examination of the literature and service user feedback.

Relevant behavioural and psychological items were added, e.g., type of sexual activity (behavioural) and perceived judgement from others regarding PrEP use (motivation). Items relevant to GBMSM were added, e.g., participant’s beliefs about how PrEP impacted their enjoyment of sex. Lastly, a question asking the length of time since the adherent/non-adherent episode was added.

### Final Measures

The full questionnaire is provided in **Appendix** 1. At the beginning of the questionnaire, demographic data and PrEP-related information, such as duration of PrEP use, were collected. Participants were able to choose whether to discuss their adherent or non-adherent episode first, reducing the risk of social desirability bias.

### Behavioural Variables

Behavioural factors for both adherent and non-adherent episodes were assessed first, to enhance episodic memory detail. These included: day of the week on which the episode occurred; how many days ago this was; whether something or someone reminded them to take their medication; the normality of the day; if they were at home or elsewhere; whether they were alone or not and if not, whether those they were with knew they used PrEP; whether they had used substances around the time of taking medication; whether they had anticipated having sex that day; if they actually had sex that day; to what extent they felt at risk from HIV without PrEP; and, if they did have sex that day, whether they used a condom, whether they’d engaged in chemsex, the HIV status of their sexual partner, type of sexual activity and positioning, and whether it was with a casual or regular sexual partner. For the non-adherent episode, participants were also asked whether non-adherence was intentional or due to forgetting, and if they had had sex that day, whether they subsequently used post-exposure prophylaxis (PEP).

### Psychological Variables

The IMB constructs were measured by questions rated on a 5-point Likert scale (very unlikely’, ‘to ‘very likely’). Each item was introduced with “At the time I was due to take my PrEP” and presented as a statement. After assessing internal consistency, the final measure had 26 items in total: three items examined information (adherent episode α = 0.79; non-adherent episode α = 0.77), thirteen items examined motivation (adherent episode α = 0.86; non-adherent episode α = 0.84) and ten items examined behavioural skills (adherent episode α = 0.85; non-adherent episode α = 0.85). This suggests each subscale had either an acceptable or good level of reliability [44].

Other situational psychological variables included positive and negative affect at the time of each episode. The same items were used as those within The International Positive and Negative Affect Schedule Short Form (I-PANAS-SF) questionnaire, which has been shown to be reliable and valid within adult populations [45]. This scale has two five-item subscales (positive and negative affect) and uses a five-point Likert scale (very slightly or not at all to extremely). The current study used the I-PANAS-SF to measure affect (adherent episode: positive affect α = 0.83, negative affect α = 0.83; non-adherent episode: positive affect α = 0.92, negative affect α = 0.85). These items were introduced with the sentence ‘How did you feel when it was time to take your PrEP?’.

### Analysis

Bivariate comparisons were conducted between psychological and behavioural variables and episodes of adherence and non-adherence. Total scores were calculated for variables measured with multiple items variables. Paired t-tests (for continuous variables that met assumptions for parametric statistics) or McNemar’s chi-squared tests (for categorical variables, using Fisher’s exact estimates for expected frequencies < 5) were used. Uncorrected McNemar values were used (i.e., without Yates correction). If normality could not be assumed for continuous variables, then paired t-tests with bootstrapping was planned. Effect sizes were calculated using Cramer’s phi (φ) for categorical variables (small effect 0.1; medium effect 0.3; large effect 0.5) and Cohen’s d for comparisons of means (small effect 0.2; medium effect 0.5; large effect 0.8) [46].

## Results

Sixty-seven participants completed the questionnaire in total. Demographic and clinical information is presented in Table 1.

**Table 1:** Demographic information

Variable		
**Age**	Mean age (SD)	37.1 (10.2)
	Median age (IQR, range)	35.4 (30–45, 18–62)
**Occupational Status**	Employed Full Time	57 (85.1%)
	Employed Part Time	4 (6.0%)
	Student Full Time	1 (1.5%)
	Student Part Time	1 (1.5%)
	Retired	1 (1.5%)
	Other	3 (4.5%)
**Highest educational qualification**	GCSE/O-level	5 (7.5%)
	A level/BTEC	11 (16.4%)
	Degree level qualification	25 (37.3%)
	Postgraduate qualification	26 (38.8%)
**Ethnicity**	White	55 (82.1%)
	Black	1 (1.5%)
	Asian	3 (4.5%)
	Mixed	4 (6.0%)
	Other	4 (6.0%)
**Born in UK?**	Yes	35 (52.2%)
	No	32 (47.8%)
**Relationship status**	Single	46 (68.7%)
	Partner, living together	15 (22.4%)
	Partner, living separately	6 (9.0%)
**Number of sexual partners (any type of sex) in the last month**	Mean (median, IQR)	8.2 (3, 2–4)

Table 2 presents descriptive PrEP-related information. The majority of participants had obtained their PrEP online, nearly all had a daily routine for taking PrEP, and most reported taking at least 4 doses in the last 7 days.

**Table 2:** Descriptive PrEP-related information

Variable		N (%)
**Length of time PrEP taken**	3–4 months	18 (27)
	5–8 months	13 (19)
	9–12 months	10 (15)
	1 year+	26 (39)
**How PrEP obtained**	Online	44 (66)
	Research/Study Participant	14 (21)
	Other	9 (13)
**Daily routine for taking PrEP e.g., after brushing teeth.**	Yes	60 (90)
	No	5 (7)
	*Missing*	*2 (3)*
**How many times PrEP taken in the last 7 days**	0	4 (6)
	1–3 doses	2 (3)
	4–6 doses	19 (28)
	7 *Missing*	40 (60) 2 (3)
**Current experience of side effects**	Yes	7 (10)
	No	60 (90)

Table 3 presents the frequencies of responses for non-adherent and adherent episodes for categorical variables. At the time of questionnaire completion, the mean number of days since the adherent episode was 2.6 days (SD = 3.05) and 11.9 days (SD = 10.06) for the non-adherent episode.

**Table 3:** Comparison of behavioural variables between adherent and non-adherent episodes (n = 67)

Variable		Adherent episode (freq. and %)	Non-adherent episode (freq. and %)	p-value^a^
**Use of Reminders**	Yes	27 (40.3)	19 (28.4)	*0.03*
	No	39 (58.2)	47 (70.1)	
	*Missing*	*1 (1.5)*	*1 (1.5)*	
**Day of the week**	Mon-Fri	55 (82.1)	43 (64.2)	*0.01*
	Sat-Sun	7 (10.4)	20 (29.9)	
	*Missing*	*5 (7.5)*	*4 (6.0)*	
**Normality of Day**	Normal	62 (92.5)	41 (57.7)	*< 0.001*
	Not normal	5 (7.5)	26 (36.6)	
	Other	0 (0.0)	4 (5.6)	
**Location**	Own home	59 (88.1)	42 (62.7)	*< 0.001*
	Somewhere else	8 (11.9)	25 (37.3)	
**Company at time of (missed) dose**	Alone	53 (79.1)	42 (62.7)	*0.02*
	Not alone	14 (20.9)	25 (37.3)	
***If not alone***: **Did person know about PrEP use?**	Yes	13 (92.9)	19 (76.0)	
	No	1 (7.1)	6 (24.0)	
**Substance use**	Yes	6 (9.0)	14 (20.9)	*0.02*
	No	59 (88.1)	51 (76.1)	
	*Missing*	*2 (3.0)*	*2 (3.0)*	
**Sex on day of (missed) dose**	Yes	21 (31.3)	15 (22.4)	0.2
	No	46 (68.7)	52 (77.6)	
***If yes to sex on day of (missed) dose***:				
**Did sexual partner know about PrEP use?**	Yes	13 (61.9)	11 (73.3)	
	No	8 (38.1)	4 (26.7)	
**Use of condom**	Yes	1 (4.8)	0 (0.0)	
	No	20 (95.2)	15 (100.0)	
**Chemsex**	Yes	3 (14.3)	5 (33.3)	
	No	18 (85.7)	10 (66.7)	
**HIV status of sexual partner**	HIV negative	7 (33.3)	7 (46.7)	
	HIV positive	5 (23.8)	3 (20.0)	
	Not known	9 (42.9)	5 (33.3)	
**Type of sex: Top**	Yes	13 (61.9)	12 (80.0)	
	No	8 (38.1)	3 (20.0)	
**Type of sex: Bottom**	Yes	13 (61.9)	8 (53.3)	
	No	8 (38.1)	7 (46.7)	
**Type of sex: Oral**	Yes	16 (76.2)	8 (53.3)	
	No	5 (23.8)	7 (46.7)	
**Type of sex: Other**	Yes	0 (0.0)	1 (6.7)	
	No	21 (100.0)	14 (93.3)	
**Casual or regular partner**	Casual	13 (61.9)	12 (80.0)	
	Regular	8 (38.1)	3 (20.0)	
**Use of PEP**	Yes	NA	2 (13.3)	
	No	NA	13 (86.7)	

^a^ Italicised results are statistically significant (p < .05)

Multiple behavioural factors were associated with PrEP non-adherence. The association was strongest for non-normality of the day (p < .001, φ = 0.48, close to large effect size) and being out of the home (p < .001, φ = 0.45, close to large effect size). Participants were also more likely to be non-adherent on weekend days (p = .01, φ = 0.33, medium effect size), when they had company (p = .02, φ = 0.29, close to medium effect size), when they were using substances (p = .02, φ = 0.29, close to medium effect size) and when they didn’t use reminders (p = .03, φ = 0.26, close to medium effect size). Non-adherence was not associated with whether they had sex on the day or not (p = .2, φ = 0.16).

Table 3. **Behavioural variables between adherent and non-adherent episodes (n = 67)**.

Descriptive statistics for psychological variables for both episodes are presented in Table 4. Lower reported information (p = .04, d = 0.28, small effect size), behavioural skills (p < .001, d = 0.44, small to medium effect size) and positive affect (p = .002, d = 0.41, close to a medium effect size) were associated with non-adherent episodes in bivariate analysis. Neither negative affect (p = .35, d = 0.12) nor motivation (p = .23, d = 0.15) were significantly associated with non-adherence.

**Table 4:** Descriptive data for psychological variables

Variable (minimum-maximum score)	Episode	Median (IQR)	Mean	SD	p-value^a^
Information (3–15)	Adherent	15 (14–15)	14.36	1.14	*0.04*
	Non-adherent	15 (13–15)	14.09	1.38	
Motivation (13–65)	Adherent	54 (47–61)	53.75	7.6	0.23
	Non-adherent	54 (48–61)	54.1	8.05	
Behavioural Skills (10–50)	Adherent	44 (40–49)	44.16	4.97	*< 0.001*
	Non-adherent	42.5 (38.25-48)	42.39	6.17	
Positive Affect (5–25)	Adherent	16 (12–20)	15.82	5.47	*0.002*
	Non-adherent	14 (9-19.5)	14.42	6.27	
Negative Affect (5–25)	Adherent	5 (5–6)	6.14	2.51	0.35
	Non-adherent	5 (5–7)	6.4	2.53	

^a^ Italicised results are statistically significant (p < .05)

## Discussion

We found that a range of situational psychological and behavioural factors helped to explain episodic PrEP adherence and non-adherence amongst GBMSM at high risk of acquiring HIV. Lower-reported information, behavioural skills and positive affect were all associated with non-adherent episodes. Multiple behavioural factors were also associated with PrEP non-adherence including non-normality of the day, being out of the home, weekend days, lack of reminders, having company, and using substances. Taken together, our results suggest that considering situational variation in psychological and behavioural factors could be of value when clinically assessing barriers to adherence and devising adherence strategies with patients.

The finding that adherence was related to higher levels of behavioural skills is consistent with research exploring the utility of the IMB model for predicting adherence between participants using ART as HIV treatment or as PrEP [35–38, 47]. Behavioural skills in our model included having the confidence and skill to self-cue and self-administer PrEP, incorporate PrEP into daily routines, and manage possible side-effects. Since our study uses a within-participant study design, the strong association we found between lower behavioural skills and episodes of non-adherence suggests that these skills can differ not only *between* participants but also *within* the same individual depending on the situation. For example, an individual may feel less confident in taking their PrEP pills if they have company. This is consistent with studies of medication adherence using a similar within-participant methodology, with young adults with perinatally-acquired HIV and adults with Beta-Thalassaemia-Major [29, 48].

Adherence was related to higher levels of PrEP information. This is supported by the IMB Model for ART adherence [49], but differs from studies which found no association between information and between-participant PrEP adherence [35–38]. In these studies, it may be that having knowledge about PrEP was a necessary but not sufficient condition for adherence, or that the items used to measure PrEP knowledge were not specific or relevant enough to PrEP adherence [35]. It is also plausible that levels of PrEP knowledge may influence adherence within-participants but not between-participant adherence depending on what specific knowledge is required to support adherence in that particular context, e.g., knowing that PrEP still works when you’ve been drinking alcohol.

Adherence was not associated with motivation. This is consistent with studies where the IMB model has been applied to between-participant adherence to both PrEP and HIV treatment [34–38, 50–52]. Furthermore, studies using a similar within-participant methodology found no relationship between motivation and adherence to treatment for HIV or Beta-Thalassaemia-Major [29, 48]. Our finding suggests that motivation may not be sufficient for PrEP adherence when adherence requires multiple behavioural skills e.g., the confidence and skill to both incorporate PrEP into daily routines and manage possible side-effects. It is possible that motivation differs *between* people but not *within* people depending on the context. On the other hand, given that PrEP access is relatively novel, many of the participants involved in this study were ‘early adopters’ who were highly motivated for PrEP use, as reflected in the motivation scores. Additionally, the majority (66%) of non-adherent episodes were reported to be due to forgetting as opposed to intentional non-adherence which may explain the lack of differences in motivation between episodes.

Non-adherence was associated with lower positive affect (e.g. “I felt attentive”) in bivariate analyses and had no association with negative affect (e.g. “I felt ashamed”). This is consistent with another within-participant study which found lower positive affect was associated with non-adherence to ART amongst HIV-positive young adults [29]. It also aligns with Van Cappellen et al. (2018) who theorise an ‘upward spiral’ framework whereby positive affect makes behaviours more likely and behaviours reinforced by positive affect are more likely to be maintained [53]. In relation to negative affect, a floor effect may have occurred as participant negative affect scores were clustered at the minimum possible score which could have resulted in a type II error [54].

Several situational behavioural variables were also associated with non-adherence, including non-normality of the day, being out of the home, weekend days, having company, using substances around the time of dosing, and lack of reminders. These findings are consistent with both between-participant and within-participant ART adherence research findings that non-adherence was associated with situational behavioural variables not included in the IMB model including a lack of routine, being out of the home and weekend days [29, 48, 55]. It is also consistent with other studies investigating between-participant PrEP adherence which found that having an established routine and use of reminders facilitated adherence whilst frequent travel, not being at home and busy lifestyles acted as barriers to PrEP adherence [21–25].

There are conflicting views in the literature as to whether substance use influences non-adherence to PrEP [56–59]. However, most of these studies examine PrEP adherence between participants. Grov et al. (2019) found that use of “club drugs” (defined as ketamine, ecstasy, gamma hydroxybutyric acid (GHB), cocaine, or methamphetamine) was not associated with adherence *between* participants, but specific events of club drug use *within* participants were significantly associated with missing a PrEP dose on the same day or the next day [60]. Other studies have also highlighted that the duration and intensity of drug use may influence adherence [22, 61]. While our study did not distinguish between the type of substance used, Grov et al. notably did not find an association between events of marijuana use or heavy drinking and PrEP adherence within participants.

We did not find an association between adherence and whether an individual had sex that day. This differs from the findings of a recent cohort study that PrEP use was associated with almost double the odds of subsequent condomless anal sex occurring [62]. Since our study focused on single events of adherence and non-adherence, this may not be representative of participants’ adherence over time.

Our findings on the association between situational behavioural variables and adherence could indicate that non-adherence is more likely when an individual’s usual routine is disrupted. When out of the home, GBMSM may not have access to PrEP and/or lack access to the usual memory cues to take their medication, making it more difficult to plan and act on the intention to adhere. This disruption in routine may be most likely at the weekend and/or when using recreational drugs or alcohol around the time of dosing. GBMSM may be less likely to take PrEP when others are present due to fear of stigmatisation as highlighted in other PrEP studies (e.g., the fear of being perceived as sexually ‘promiscuous’ or HIV-positive) [21, 25, 26, 63]. Overall, differences in behavioural factors between adherent and non-adherent episodes suggest that contexts and lifestyle factors which may vary over time may influence PrEP adherence.

### Limitations

This study was limited by the use of non-standardised situational measures, which require further validation in larger samples. The participants were recruited from two London sexual health clinics, meaning our findings may have limited generalisability to other geographical areas or people with similar access to healthcare services. Participants were highly educated, most actively sought PrEP through online methods and had high levels of self-reported adequate adherence (4 + doses adhered to in last 7 days). This suggests that participants may have been a highly motivated sample leading to selection bias. Participants also received increased follow-up by nature of participating in the Impact or DISCOVER studies which may limit generalisability. This means that the predictors of PrEP adherence identified in the current study may differ among the wider GBMSM population and other populations using PrEP, such as transgender women and heterosexuals at elevated risk of HIV.

Our findings are also limited to those using a daily dosing regimen. However, since approximately 75% of PrEP users were using a daily dosing regimen in 2019 [64], we believe they remain relevant to the majority of PrEP users.

The questionnaire asked participants about one taken and missed PrEP dose in the previous month. These episodes may not be representative of the individual’s PrEP adherence episodes as those reported on may have been more memorable for particular reasons. Since the non-adherent episodes were, on average, substantially longer away in time than adherent episodes, this may have resulted in recall bias. However, a PrEP implementation study conducted in 2017 which measured adherence through biological and self-report methods suggested that GBMSM can provide accurate self-report data over a 30-day period [65].

Although participants were asked about sex on the day of the (missed) dose, there was also no attempt to assess whether PrEP non-adherence amounted to insufficient coverage during exposure to HIV risk. Notably, only 9% of respondents this study reported having taken < 4 doses of PrEP in the last 7 days, and predictors of adherence may differ depending on whether prevention-effective adherence has already been achieved.

Finally, 66% of non-adherent episodes were reported to be due to forgetting as opposed to intentional non-adherence. There may be different predictors between intentional and non-intentional non-adherence, as indicated by previous research exploring ART adherence among adults living with HIV [66]. Further (and in contrast to HIV treatment), as 4 doses a week are sufficient for maintaining prevention-effective adherence, intentional non-adherence may remain in line with clinical PrEP guidance [67].

### Recommendations for Future Research

Further research with larger samples and data on both medication use and potential risk exposure is needed to understand different predictors of PrEP adherence within individuals who achieved prevention-effective adherence and individuals who did not, and those who intentionally and unintentionally missed doses. Future PrEP adherence research should also examine PrEP adherence in a diversity of key populations and sub-populations, settings, dosing regimens, and PrEP delivery methods to increase the generalisability of findings and delineate specific facilitators and barriers to PrEP use. Prospective studies using ecological momentary assessments of adherence episodes (e.g., using smart-app technology to ask individuals questions about episodes through daily text) would reduce the reliance upon memory and could enhance measurement reliability and validity [68, 69]. This would also allow more than one episode to be measured and may give a more representative picture of PrEP adherence.

Our findings give support to some aspects of the IMB model to help explain PrEP adherence but also highlight predictors related to PrEP adherence which are not acknowledged within the model (i.e., situational behavioural factors such as location, or positive affect). Future research should incorporate but not be limited to this theoretical model when deciding which predictors to investigate in relation to PrEP adherence.

### Implications for Clinical Practice

The findings suggest that across situations people may need different PrEP information or behavioural skills to adhere. For example, if outside of their own home, it may be particularly important to self-cue the administration of their PrEP medication. Clinical guidelines and practitioners should note that even mostly adherent PrEP users may have episodes of non-adherence and therefore all clients should regularly be counselled on adherence. Tools to support this could range from simple methods like setting an alarm on their mobile phone, to more sophisticated wrist-worn devices which use sensor-based technologies to trigger reminders [70]. Alternatively, in a situation where alcohol or other drugs are present, an individual may require information on the influence of alcohol and drugs on remembering to take a PrEP dose (whereas in other situations this may not be relevant).

Clinically, the finding that modifiable psychological and behavioural factors are important for adherence suggests that assessments of facilitators and barriers of PrEP adherence could focus upon situational variations in information, positive affect, behavioural skills, and behavioural factors (e.g., location, day of the week). Studies of PrEP adherence support indicates that therapeutic approaches combining techniques from problem solving therapy and cognitive behavioural therapy hold promise for reducing inconsistent adherence [71–73]. Such an approach could be adapted to conceptualise high-risk situations for non-adherence, working with the PrEP user to construct alternative strategies to facilitate adherence in these situations. Advanced planning could take the form of developing implementation intentions, whereby an individual would plan when and how they enact PrEP adherence in specific situations [74]. If individuals struggle to adhere in particular contexts, they may benefit from alternative modes of PrEP delivery such as long-acting injectable cabotegravir, although retention support will still be required.

### Electronic Supplementary Material

Below is the link to the electronic supplementary material.

Supplementary Material 1

## Data Availability

Anonymised electronic data from the study is stored in the RHUL Research Data Archive. This data is freely accessible under Creative Commons CC BY licence and will be retained for 10 years.

## Footnotes

1 As opposed to on-demand dosing. Daily dosing was an inclusion criterion because it was the only WHO-recommended dosing regimen at the time of the study and as the inclusion of multiple dosing regimens would have risked compromising statistical analysis if insufficient participants were recruited.
