# Supplementary material for: Psychological and Behavioural Within-participant Predictors of Adherence to Oral HIV Pre-Exposure Prophylaxis (PrEP)
Source: AIDS Behav. 2023 Aug 14;28(1):274–84. doi: 10.1007/s10461-023-04151-8 (PMC10803569; doi:10.1007/s10461-023-04151-8)
Supplement: Supplementary file 1 — Supplementary Material 1 [file 10461_2023_4151_MOESM1_ESM.docx]

# Appendices

## Appendix 1

**
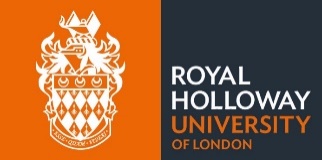
**

**PrEP Situational Adherence Questionnaire**

**Demographic Information:**

Your date of birth **(DD/MM/YYYY) format**:

……………………..

Occupational status **(please tick):**

| Employed Full Time | ☐ | Employed Part Time | ☐ | Unemployed | ☐ | Student  Full Time | ☐ | Student Part Time | ☐ |
| --- | --- | --- | --- | --- | --- | --- | --- | --- | --- |
| Retired | ☐ | Other | ☐ |  |  |  |  |  |  |

Highest educational qualification **(please tick):**

| Not Applicable | ☐ | GCSE /O-level | ☐ | A-level/ BTEC | ☐ | Degree level Qualification | ☐ | Postgraduate Qualification | ☐ |
| --- | --- | --- | --- | --- | --- | --- | --- | --- | --- |

Ethnicity **(please tick):**

| White | ☐ | Black | ☐ | Asian | ☐ | Mixed | ☐ | Other | ☐ |
| --- | --- | --- | --- | --- | --- | --- | --- | --- | --- |

If other, **please specify:**

………………………….

Born in the UK? **(please tick):**

| Yes | ☐ | No | ☐ |
| --- | --- | --- | --- |

Relationship status **(please tick):**

| Single | ☐ |
| --- | --- |
| Partner, living together | ☐ |
| Partner, living separately | ☐ |

Number of sexual partners in the last month **(please write):**

…………………..

How long have you been taking PrEP for? **(please tick):**

| 3-4 months | ☐ | 5-8 months | ☐ | 9-12 months | ☐ | 1 year+ | ☐ |  |
| --- | --- | --- | --- | --- | --- | --- | --- | --- |
| How do you obtain your PrEP medication? (please tick):   \| Online \| ☐ \| Private Prescription \| ☐ \| Research/Study Participant \| ☐ \| Friend \| ☐ \| Other \| ☐ \| \| --- \| --- \| --- \| --- \| --- \| --- \| --- \| --- \| --- \| --- \|   If ‘other’ please specify where you obtain PrEP:  …………………    Do you have a daily routine for when/how you take your PrEP? (please tick)   \| Yes \| ☐ \| No \| ☐ \| \| --- \| --- \| --- \| --- \|   What clinic do you attend for PrEP monitoring? (please tick):   \| Site 1 \| ☐ \| Site 2 \| ☐ \| Site 3 \| ☐ \| \| --- \| --- \| --- \| --- \| --- \| --- \|   Following your daily dosing regimen, how many times have you taken your medication in the last 7 days? (please write):  ………………….  Are you experiencing side effects? (please tick):   \| Yes \| ☐ \| No \| ☐ \|  \|  \| \| --- \| --- \| --- \| --- \| --- \| --- \|   If Yes, are these side effects distressing you? (please tick):   \| Yes \| ☐ \| No \| ☐ \| N/a \| ☐ \| \| --- \| --- \| --- \| --- \| --- \| --- \|   *Please take a moment to think about a time when you did take your PrEP. For example, think about where you were, how you were feeling, what you expected to do that day or who you expected to see.* | | | | | | | |  |

Think about the time when you **did take** your PrEP.

Please answer these questions about what you thought and how you felt at that time.

What day of the week was it **(when you did take your PrEP)?:**

…………………………………….

Please write how many days ago this was **(when you did take your PrEP)** e.g. 2 days ago, 3 days ago etc?:

…………………………………

Was there someone there or did you have other prompts e.g. pill box or alarm to remind you to take your PrEP at the time? **(please circle):**

Yes No

Please tick which one applied to you at the time **(when you did take your PrEP) (please tick):**

My day was the same as normal ☐

My day was different to normal because of something unexpected ☐

My day was different to normal because I had made plans ☐

Other ☐

Where were you? **(when you did take your PrEP) (please tick):**

Own home ☐ A friend’s house ☐ Partner’s house ☐

A public place (e.g. work or college) ☐ Somewhere else ☐

If you ticked ‘somewhere else’ **please specify:**

…………………………………………………….

Who were you with? (when it was time to take your PrEP and you did take it) **(please tick):**

Alone ☐ With a friend ☐ With a partner ☐ With family ☐

With an acquaintance ☐ With a work colleague ☐ Someone else ☐

If you ticked ‘someone else’ **please specify:**

…………………………………………………….

If you weren’t alone, did this person/these people know you were taking PrEP? **(please circle):**

Yes No N/a

Were you using alcohol or taking drugs (e.g. cannabis, ecstasy, G, crystal meth/Tina, mephedrone) around the time you took your PrEP? **(please circle):**

Yes No

How likely did you think it was that you were going to have sex on that day? **(please circle):**

| Very Unlikely | Unlikely | Neither likely or unlikely | Likely | Very Likely |
| --- | --- | --- | --- | --- |
| 1 | 2 | 3 | 4 | 5 |

Did you have sex on that day? **(please circle):**

Yes No

When you were due to take your medication, to what extent did you feel you were at risk of HIV without taking PrEP? **(please circle):**

| Very Unlikely | Unlikely | Neither likely or unlikely | Likely | Very Likely |
| --- | --- | --- | --- | --- |
| 1 | 2 | 3 | 4 | 5 |

If you did have sex on that day, did the person you had sex with know you were taking PrEP? **(please circle):**

Yes No N/a

If you did have sex on that day, did you use a condom? **(please circle):**

Yes No N/a

If you did have sex on that day, was this chemsex?  **(please circle):**

Yes No N/a

If you did have sex on that day, what was the status of your sexual partner **(please circle):**

HIV negative HIV positive Don’t know N/a

If you did have sex on that day, was this **(please circle one or more):**

Anal insertive (top) Anal receptive (bottom) Oral sex

Other N/a

If you did have sex on that day, was this with a casual or regular partner? **(please circle):**

Casual partner Regular partner N/a

*Please again, take a moment to think about a time when* ***you did*** *take your PrEP. For example, think about where you were, how you were feeling, what you expected to do that day or who you expected to see.*

**At the time I was due to take my PrEP…**

|  | 1 Strongly disagree | 2 Disagree | 3 Neither agree or disagree | 4 Agree | 5 Strongly agree |
| --- | --- | --- | --- | --- | --- |
| I knew the correct way to take my medicines |  |  |  |  |  |
| I knew how taking PrEP could make me feel |  |  |  |  |  |
| I understood how my PrEP would work |  |  |  |  |  |
| I knew I should take the PrEP at that time |  |  |  |  |  |
| I thought other people would notice I was taking my PrEP and think I was HIV positive which concerned me |  |  |  |  |  |
| I thought that other people would notice me taking PrEP and think I was promiscuous, which bothered me |  |  |  |  |  |
| I knew how to tell others about my PrEP use |  |  |  |  |  |
| I thought I had to plan my life around my medicine, which frustrated me |  |  |  |  |  |
| PrEP reminded me I was at risk of HIV, which bothered me |  |  |  |  |  |
| I felt confident that my PrEP was going to work |  |  |  |  |  |
|  | 1 Strongly disagree | 2 Disagree | 3 Neither agree or disagree | 4 Agree | 5 Strongly agree |
| People around me that I care about were supportive about my PrEP |  |  |  |  |  |
| I thought that I would have to take these medicines every day during the time I was at risk, which I did not like |  |  |  |  |  |
| I thought my PrEP might interact with other substances/drugs I was taking, which bothered me |  |  |  |  |  |
| I thought that taking PrEP might stop me from taking precautions against STI’s, which bothered me |  |  |  |  |  |
| I thought that taking PrEP was easier than using a condom |  |  |  |  |  |
| I thought PrEP would make me worry less about HIV |  |  |  |  |  |
| I thought PrEP would make me enjoy sex more |  |  |  |  |  |
| I thought PrEP was harming me |  |  |  |  |  |
| I thought PrEP would cause side effects |  |  |  |  |  |
| I had easy access to my medicines |  |  |  |  |  |
|  | 1 Strongly disagree | 2 Disagree | 3 Neither agree or disagree | 4 Agree | 5 Strongly agree |
| I was confident I could find the time to take my PrEP |  |  |  |  |  |
| I was confident I could manage any side effects |  |  |  |  |  |
| I was confident that I could remember to take my medicines |  |  |  |  |  |
| I was confident I could manage the size of the pills or the taste of the medicine |  |  |  |  |  |
| I felt confident that I had enough PrEP for other days |  |  |  |  |  |
| I felt confident that I could fit my medicines around what I was doing |  |  |  |  |  |
| I felt confident I could take my medicines correctly |  |  |  |  |  |
| I felt confident I could take my medicines even if other people were around |  |  |  |  |  |
| I felt confident I could ask for help to take my PrEP if I needed to |  |  |  |  |  |
| I felt confident I could take my medicines however I was feeling |  |  |  |  |  |
| I felt ill |  |  |  |  |  |
|  |  |  |  |  |  |

How did you feel when it was time to take your PrEP?

|  | 1  Very slightly or Not at all | | 2  A little | 3  Moderately | | 4  Quite a bit | 5 Extremely |
| --- | --- | --- | --- | --- | --- | --- | --- |
| Active |  |  | | |  |  |  |
| Afraid |  |  | | |  |  |  |
| Determined |  |  | | |  |  |  |
| Nervous |  |  | | |  |  |  |
| Attentive |  |  | | |  |  |  |
| Upset |  |  | | |  |  |  |
| Inspired |  |  | | |  |  |  |
| Hostile |  |  | | |  |  |  |
| Alert |  |  | | |  |  |  |
| Ashamed |  |  | | |  |  |  |
|  |  |  | | |  |  |  |
|  |  |  | | |  |  |  |
|  |  |  | | |  |  |  |
|  |  |  | | |  |  |  |
|  |  |  | | |  |  |  |
|  |  |  | | |  |  |  |
|  |  |  | | |  |  |  |
|  |  |  | | |  |  |  |


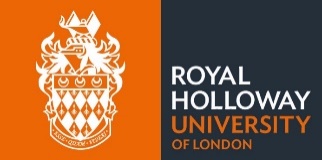


**PrEP Situational Adherence Questionnaire**

*Now please take a moment to think about a time when* ***you did not*** *take your PrEP. For example, think about where you were, how you were feeling, what you expected to do that day or who you expected to see.*

Think about the time when you **did not take** your PrEP.

Please answer these questions about what you thought and how you felt at that time.

What day of the week was it **(when you did not take your PrEP)?:**

…………………………………….

Please write how many days ago this was **(when you did not take your PrEP)** e.g. 2 days ago, 3 days ago etc?:

…………………………………

Did you forget to take your PrEP or did you intentionally not take your medication? **(please tick):**

I forgot ☐ I chose to not take my medication ☐

If you chose not to take your PrEP please write why:

………………………………………………………………………………………………….

Was there someone there or did you have other prompts e.g. pill box or alarm to remind you to take your PrEP at the time? **(please circle):**

Yes No

Please tick which one applied to you at the time **(when you did not take your PrEP):**

My day was the same as normal ☐

My day was different to normal because of something unexpected ☐

My day was different to normal because I had made plans ☐

Other ☐

Where were you? **(when you did not take your PrEP) (please tick):**

Own home ☐ A friend’s house ☐ Partner’s house ☐

A public place (e.g. work or college) ☐ Somewhere else ☐

If you ticked ‘somewhere else’ **please specify:**

…………………………………………………….

Who were you with? **(**when it was time to take your PrEP and you did not take it) **(please tick):**

Alone ☐ With a friend ☐ With a partner ☐ With family ☐

With an acquaintance ☐ With a work colleague ☐ Someone else ☐

If you ticked ‘someone else’ **please specify:**

…………………………………………………….

If you weren’t alone, did this person/these people know that you were taking PrEP? **(please circle):**

Yes No N/a

Were you using alcohol or taking drugs (e.g. cannabis, ecstasy, G, crystal meth/Tina, mephedrone) around the time you were meant to take your PrEP? **(please circle):**

Yes No

How likely did you think it was that you were going to have sex on that day?  **(please circle):**

| Very Unlikely | Unlikely | Neither likely or unlikely | Likely | Very Likely |
| --- | --- | --- | --- | --- |
| 1 | 2 | 3 | 4 | 5 |

Did you have sex on that day? **(please circle):**

Yes No

When you were due to take your medication, to what extent did you feel you were at risk of HIV? **(please circle):**

| Very Unlikely | Unlikely | Neither likely or unlikely | Likely | Very Likely |
| --- | --- | --- | --- | --- |
| 1 | 2 | 3 | 4 | 5 |

If you did have sex on that day, did this person you had sex with know you were taking PrEP? **(please circle):**

Yes No N/a

If you did have sex on that day, did you use a condom? **(please circle):**

Yes No N/a

If you did have sex on that day, did you use **post**-exposure prophylaxis (i.e. PEP medication)? **(please circle):**

Yes No N/a

If you did have sex on that day, was this chemsex?  **(please circle):**

Yes No N/a

If you did have sex on that day, what was the status of your sexual partner **(please circle):**

HIV negative HIV positive Don’t know N/a

If you did have sex on that day, was this **(please circle one or more):**

Anal insertive (top) Anal receptive (bottom) Oral sex

Other N/a

If you did have sex on that day, was this with a casual or regular partner? **(please circle):**

Casual partner Regular partner N/a

*Please again, take a moment to think about a time when* ***you did not*** *take your PrEP medication. For example, think about where you were, how you were feeling, what you expected to do that day or who you expected to see.*

**At the time I was due to take my PrEP…**

|  | 1 Strongly disagree | 2 Disagree | 3 Neither agree or disagree | 4 Agree | 5 Strongly agree |
| --- | --- | --- | --- | --- | --- |
| I knew the correct way to take my medicines |  |  |  |  |  |
| I knew how taking PrEP could make me feel |  |  |  |  |  |
| I understood how my PrEP would work |  |  |  |  |  |
| I knew I should take the PrEP at that time |  |  |  |  |  |
| I thought other people would notice I was taking my PrEP and think I was HIV positive which concerned me |  |  |  |  |  |
| I thought that other people would notice I was taking my PrEP and think I was promiscuous which bothered me |  |  |  |  |  |
| I knew how to tell others about my PrEP use |  |  |  |  |  |
| I thought I had to plan my life around my medicine, which frustrated me |  |  |  |  |  |
| PrEP reminded me I was at risk of HIV, which bothered me |  |  |  |  |  |
| I felt confident that my PrEP was going to work |  |  |  |  |  |
|  | 1 Strongly disagree | 2 Disagree | 3 Neither agree or disagree | 4 Agree | 5 Strongly agree |
| People around me that I care about were supportive about my PrEP |  |  |  |  |  |
| I thought that I would have to take these medicines every day during the time I was at risk, which I did not like |  |  |  |  |  |
| I thought my PrEP might interact with other substances/drugs I was taking, which bothered me |  |  |  |  |  |
| I thought that taking PrEP might stop me from taking precautions against STI’s, which bothered me |  |  |  |  |  |
| I thought that taking PrEP was easier than using a condom |  |  |  |  |  |
| I thought PrEP would make me worry less about HIV |  |  |  |  |  |
| I thought PrEP would make me enjoy sex more |  |  |  |  |  |
| I thought PrEP was harming me |  |  |  |  |  |
| I thought PrEP would cause side effects |  |  |  |  |  |
| I had easy access to my medicines |  |  |  |  |  |
|  | 1 Strongly disagree | 2 Disagree | 3 Neither agree or disagree | 4 Agree | 5 Strongly agree |
| I was confident I could find the time to take my PrEP |  |  |  |  |  |
| I was confident I could manage any side effects |  |  |  |  |  |
| I was confident that I could remember to take my medicines |  |  |  |  |  |
| I was confident I could manage the size of the pills or the taste of the medicine |  |  |  |  |  |
| I felt confident that I had enough PrEP for other days |  |  |  |  |  |
| I felt confident that I could fit my medicines around what I was doing |  |  |  |  |  |
| I felt confident I could take my medicines correctly |  |  |  |  |  |
| I felt confident I could take my medicines even if other people were around |  |  |  |  |  |
| I felt confident I could ask for help to take my PrEP if I needed to |  |  |  |  |  |
| I felt confident I could take my medicines however I was feeling |  |  |  |  |  |
| I felt ill |  |  |  |  |  |
|  |  |  |  |  |  |

How did you feel when it was time to take your PrEP?

|  | 1  Very slightly or Not at all | | 2  A little | 3  Moderately | | 4  Quite a bit | 5 Extremely |
| --- | --- | --- | --- | --- | --- | --- | --- |
| Active |  |  | | |  |  |  |
| Afraid |  |  | | |  |  |  |
| Determined |  |  | | |  |  |  |
| Nervous |  |  | | |  |  |  |
| Attentive |  |  | | |  |  |  |
| Upset |  |  | | |  |  |  |
| Inspired |  |  | | |  |  |  |
| Hostile |  |  | | |  |  |  |
| Alert |  |  | | |  |  |  |
| Ashamed |  |  | | |  |  |  |
|  |  |  | | |  |  |  |
|  |  |  | | |  |  |  |
|  |  |  | | |  |  |  |
|  |  |  | | |  |  |  |
